# Serbian Health Information System (HIS) improvements 2021–2024: comparison study using stages of continuous improvement (SOCI) methodology

**DOI:** 10.1186/s12961-025-01337-5

**Published:** 2025-07-14

**Authors:** Bosiljka Djikanovic, Milan Kovacevic, Isidora Smigic, Marija Cvejic, Emilija Nicic, Caitlin Madevu-Matson, Steve Ollis, Vasa Curcin, Alimou Barry, Stephanie Watson-Grant

**Affiliations:** 1 Institute of Social Medicine, Faculty of Medicine, University of Belgrade, Subotica 8, 11000 Belgrade, Serbia; 2 Centre - School of Public Health, Faculty of Medicine, University of Belgrade, Subotica 8, 11000 Belgrade, Serbia; 3 JSI, Country Health Information Systems and Data Use (CHISU), Arlington, United States of America; 4 National Alliance for Local Economic Development NALED, Belgrade, Serbia; 5 Department of Population Health Sciences, School of Life Course & Population Sciences, Faculty of Life Sciences and Medicine, King’s College London, London, United Kingdom

**Keywords:** Health information systems, Health policy, Health systems plans, Serbia, Assessment, eHealth, Consensus, Information technology

## Abstract

**Background:**

The Health Information System (HIS) in public healthcare services in Serbia was introduced in 2008, with the first comprehensive evaluation of its maturity conducted in 2021. Since then, several improvement initiatives have been implemented. This study aimed to assess the extent of HIS advancement between 2021 and 2024 and to identify both the desirable and realistic future maturity status.

**Methods:**

The maturity assessment of the Serbian HIS in 2024 was conducted using the same tool as in 2021: The Health Information Systems Stages of Continuous Improvement (SOCI), enabling direct comparison between the two periods. Progress was measured across five domains: Leadership and Governance, Management and Workforce, Information and Communication Technologies (ICT), Standards and Interoperability, and Data Quality and Use. These domains covered 13 components and 39 single subcomponents, with their maturity stages being assessed on a 5-point Likert scale on the basis of the opinions of key informants and documented through a desk review. Higher scores indicate a higher level of development. Along with a current assessment of maturity, key informants identified desired maturity levels for the future, using the same scale. Data were presented as comparisons in total scores per domain in 2024 versus 2021, for both current and projected statuses.

**Results:**

Between 2021 and 2024, the overall maturity of the Serbian HIS improved by nearly 1 point (from 1.6/5 to 2.5/5). The same difference of 0.9 was observed between the current 2024 status and the future desired status (2.5 versus 3.4). The most notable improvements were observed in the HIS Strategic Plan under Leadership and Governance (2.5-point increase) and Business Continuity under ICT Infrastructure (2-point increase). The primary driver of progress over the past 3 years was the adoption of the national Program for Digitalization in the Health System of Serbia (eHealth Strategy) and its corresponding Action Plan, which served as a development blueprint.

**Conclusions:**

Substantial progress in HIS maturity was achieved between 2021 and 2024, driven by strong governmental commitment, international donor support, and the engagement of dedicated national professionals. If current momentum and resourcing are sustained, the projected maturity levels are likely to be attainable in the near future.

**Supplementary Information:**

The online version contains supplementary material available at 10.1186/s12961-025-01337-5.

## Background

**Fig. 1:**
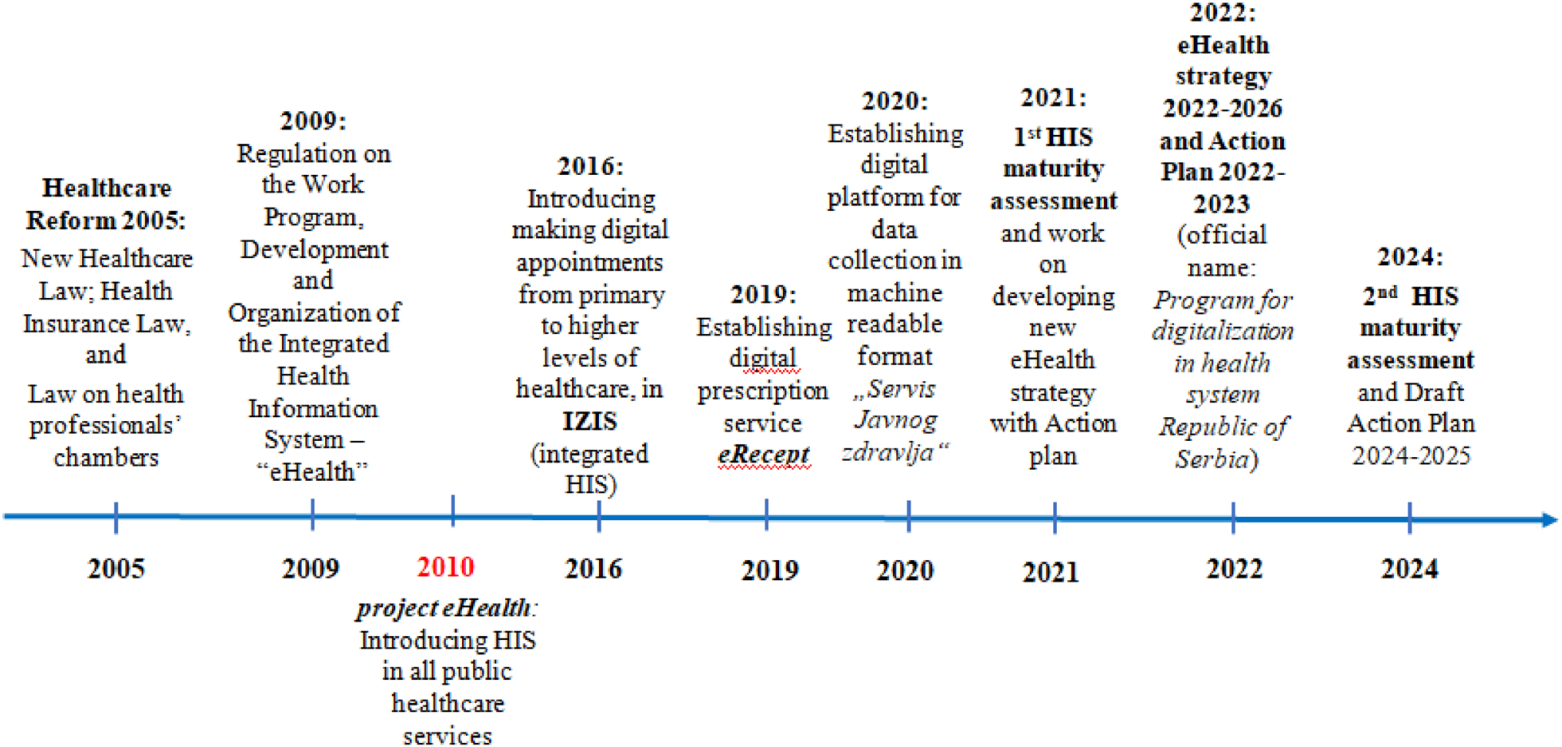
Milestones of incremental achievements of HIS in Serbia since 2005. Source: updated and modified from Ollis et al., 2024

The Health Information System (HIS) plays a key role in improving the administration and provision of health services, supporting more efficient management of resources, increasing transparency and accountability in a country’s health system, and improving quality of care [1, 2]. In the Republic of Serbia, the HIS was gradually introduced in all public health facilities beginning in 2010. The initial goal was to establish e-health records, with assumed patient consent, unless individuals explicitly refused. In addition, later on, HIS expanded its functionalities and interoperability, such as enabling general practitioners (GPs) to electronically schedule appointments at a higher level of healthcare (in 2016); prescribing medicines to patients and electronically placing an order to pharmacies (in 2019); and establishing a digital platform for data collection in a machine readable format (in 2020) (Fig. 1). With the rapid advancement of information technology (IT) in recent years, the HIS in Serbia has gained increased governmental attention, leading to a new wave of comprehensive improvements [3, 4].

Serbia is a middle-income country located in the Western Balkans, commonly referred to as part of South-Eastern Europe [5]. It has an estimated population of 6,623,183 (excluding data for Kosovo and Metohija) [6]. The healthcare system operates through a comprehensive network of public healthcare institutions, delivering services at primary, secondary, and tertiary levels [7, 8]. Nationwide, approximately 350 state-owned healthcare entities are funded by the one and only statutory Health Insurance Fund (HIF), which operates on the Bismarck principles of solidarity [9]. While HIF is in charge of financing ongoing expenditures and costs related to provision of healthcare, including arrangements with providers of HIS, infrastructural improvements such as technical renovations, larger investments in buildings and purchasing new technical equipment are often financed from the credits/loans or donations, from the international organizations such as World Bank, International Monetary Fund, the European Union and international development agencies of developed countries.

Assessing the maturity status of a national HIS is a critical step towards strengthening health system performance since it enables systematic identification of strengths, gaps, and priority areas for development and supports evidence-based planning and investment in health information infrastructure [1, 10, 11]. It facilitates a benchmarking process, promotes interoperability and ensures alignment with international standards and recommended digital health strategies [10]. Furthermore, maturity assessments enhance readiness for data-driven decision-making, improve coordination with development partners and contribute to greater resilience and responsiveness in health emergencies. As such, they represent an essential tool for advancing the quality, efficiency and sustainability of health systems.

The first assessment of the maturity level of the HIS was conducted in 2021, and it ended in a 15-page internal report that was shared with key stakeholders and contributed to the development of the eHealth Strategy and Action Plan, as described in Ollis et al. [4]. The maturity assessment was conducted within the process of development of the national program for digitalization (also known as eHealth Strategy), which was run by the working group appointed by the government. The methodology used for maturity assessment was *Health Information Systems Stages of Continuous Improvement (SOCI)*, supported by the Country Health Information Systems and Data Use (CHISU) program, a 5-year United States Agency for International Development (USAID)-funded initiative (2020–2025), focused on strengthening health information systems in more than 20 countries worldwide [10]. In 2021, the maturity assessment of HIS in Serbia, using the SOCI tool, indicated that overall development was 1.6 out of 5, which is the overall status between the so-called emerging and ad hoc stage, i.e. beginning of development [4].

Periodic evaluation of the level of maturity of the HIS can help in monitoring progress in HIS development, identifying the current state and challenges, defining the future direction and courses of action, and prioritizing activities in further development [10]. A strong HIS should be well-defined, comprehensive, functional, adaptable, resilient and scalable. The system should be able to collect, manage, analyse and disseminate health data in a timely manner so that decision-makers and managers can cope with the prompt solutions, adequate resource allocation and both short-term and long-term strategic planning and organization [11].

This study aimed to determine the level of HIS maturity in 2024 and compare it with the results in 2021, i.e. what was achieved, how progress was assessed and what should be a realistic pace and expected development in the upcoming 3–4 years. Since many strategic and normative changes have happened in the previous 3 years in Serbia, such as the adoption of a national eHealth Strategy and the Law on Health Documentation and Records, we hypothesized that it positively affected HIS advancements, which will be reflected in an increase in the overall maturity score as well.

## Methods

### The instrument

The 2024 assessment of HIS maturity was conducted using the same tool that was applied in 2021: the Health Information Systems Stages of Continuous Improvement (SOCI) [10]. This tool, developed by MEASURE Evaluation through USAID funding and collaboration with the WHO, Centers for Disease Control and Prevention (CDC) and the Health Data Collaborative, has been successfully implemented in several low- and middle-income countries (Uganda, Ethiopia, Niger, Madagascar, Indonesia, Burkina Faso, Malawi, Timor Leste and Burundi) as a part of the process of enhancing their HIS [12–19]. The SOCI toolkit provides a structured framework for assessing HIS maturity at the national level across five core domains: Leadership and Governance; Management and Workforce; Information and Communication Technologies; Standards and Interoperability; and Data Quality and Use [10]. These domains are further divided into 13 HIS components and 39 subcomponents. Each subcomponent is evaluated using a 5-point ordinal Likert scale, with the following interpretations: 1 – emerging/ad hoc status; 2 – repeatable; 3 – defined; 4 – managed; and 5 – optimized. A score of 5 represents the highest level of maturity, while a score of 1 indicates the lowest level. Each of these points on the scale of 1–5, for each of the 39 subcomponents, had its own specific brief description, up to three sentences, which were translated to Serbian (Supplementary File 1). The assessment in 2024 was conducted in several steps depicted below. 

### The process of maturity assessment

#### Step 1. Establishing the core team

The core assessment team consisted of the members of the organization National Alliance for Local Economic Development (NALED), who were in charge of continuous monitoring of the implementation of The Digitalization Program in the Health System of the Republic of Serbia from 2022 to 2026 (later in the text: eHealth Strategy) and eHealth Action Plan 2022–2023 [19, 20]; an independent expert (leading evaluator); and staff from the CHISU Serbia project team. All core team members were involved in the evaluation in 2021, so they were already familiar with the methodology and the process (4). 

#### Step 2. Defining the scope and assessment approach

The core team decided that the optimal approach to maturity assessment would be conducting a desk review, proposing the initial maturity status (points on a 1–5 scale described above) per subcomponent by core team (Step 3) and citing available documents from desk review that justify given points. This is the same approach that was taken in 2021 (4). After identifying key domain experts (Step 4), initial points were validated, reviewed and discussed in group interviews with those experts, which is described in depth in Step 5.

#### Step 3. Carrying out landscape analysis and desk reviews

In the initial phase of this step, we mapped 39 subcomponents of the SOCI tool to the 99 objectives and tasks of the eHealth Action Plan 2022–2023 to identify whether elements of the SOCI tool (39 subcomponents) were addressed in the eHealth Action Plan [10, 21]. The desk review also identified the status of the tasks in the eHealth Action Plan, through an internal monitoring document maintained by NALED, which informed and justified initially given points [22]. Points in the near future (upcoming 3–4 years) were given on the basis of a draft eHealth Action Plan 2024–2026. In the case that SOCI subcomponents or their proxies (items or activities with similar interpretation) were not identified in the eHealth Action Plan 2022–2023, a broader perspective in the assessment of that component was used, and other legislative and strategic documents that might be relevant for that particular subcomponent were considered, analysed and reported. The same as in the assessment in 2021, examples of reviewed documents in 2024 included legislation acts, strategies and official reports issued by public health institutions and similar official institutions (4). The results of this landscape analysis and desk review were thoroughly documented in a draft SOCI report, which was sent to domain experts ahead of the interviews, for their considerations in terms of validations of proposed points, comments or suggestions.

#### Step 4: Selecting domain experts (key informants)

The core assessment team identified national domain experts who were key informants for validation of the results of the desk review and assigned points on the basis of the following criteria: extensive knowledge in a designated SOCI domain and extensive national or institutional experience in the implementation of IT solutions in the designated domain in the last period. Both criteria needed to be fulfilled. A total of six domain experts were involved in the SOCI assessment in 2024, and they were employed in the following institutions: Institute of Public Health of Serbia “Dr Milan Jovanovic Batut”, The Office for IT and Government, Agency for Medicines and Medical Devices (ALIMS), NALED and Centre for Fourth Industrial Revolution in Belgrade, Serbia [23–27]. Their involvement was anonymous and voluntary, and they provided consent for recording group interviews (Step 5).

#### Step 5: Organizing group interviews

In the fifth step, the core assessment team contacted domain experts and scheduled a meeting/interview (these two terms will be used interchangeably in this paper), with an aim to discuss initial findings, i.e. description of the current status of each single subcomponent and assigned points for current status. In addition, the team also assigned points for the desired status over the next 3–4 years. Prior to the meeting, experts were emailed the initial draft report, along with the short description of the points on a scale of 1–5, per each subcomponent, as translated from the SOCI tool (Supplementary File 1). Each domain expert was typically assigned to one or two domains. The interviews were conducted online by two or three core team members who took notes and made ad hoc changes to the report and scores on the basis of the feedback of experts and mutual discussion. It is important to emphasize that during interviews, both the core assessment team (interviewers) and key informants did not have results of the SOCI assessment from 2021 in front of them, which might have influenced assessment.

After the meeting and adjusting the content of the report according to experts’ suggestions, domain experts received an updated version of a report for another cycle of validation (approval) of changes, and/or additional corrections and suggestions. Typically, experts’ opinions differed by no more than 1 point, and in the case of that occurring, a thorough reading of the description of the points took place at the meeting, and through discussions, experts harmonized their opinions on a final consensus score.

After completing all planned interviews and revisions of the report, all domain experts received the final draft version of the report and were asked to review it for comments and suggestions in all domains. Therefore, the final draft version of a report is a result of a consensus achieved in three iterations of group work, which is basically the same process that was carried out in 2021. The final draft report and the conclusions were presented at the project close-out meeting in September 2024, during which no additional changes were made.

### Statistical analysis

The outcome of the consensus method was 1 single point assigned to each of the 39 subcomponents on a 5-point ordinal Likert scale, with the following interpretation of points: (1) emerging/ad hoc; (2) repeatable; (3) defined; (4) managed; and (5) optimized. Scores per each of the five domains and 13 components within domains were calculated as an average of points per component (and per domain), rounded to one decimal place. Results from the evaluation of both current and future maturity assessments in 2024 were compared with 2021 and graphically presented.

## Results

**Fig. 2:**
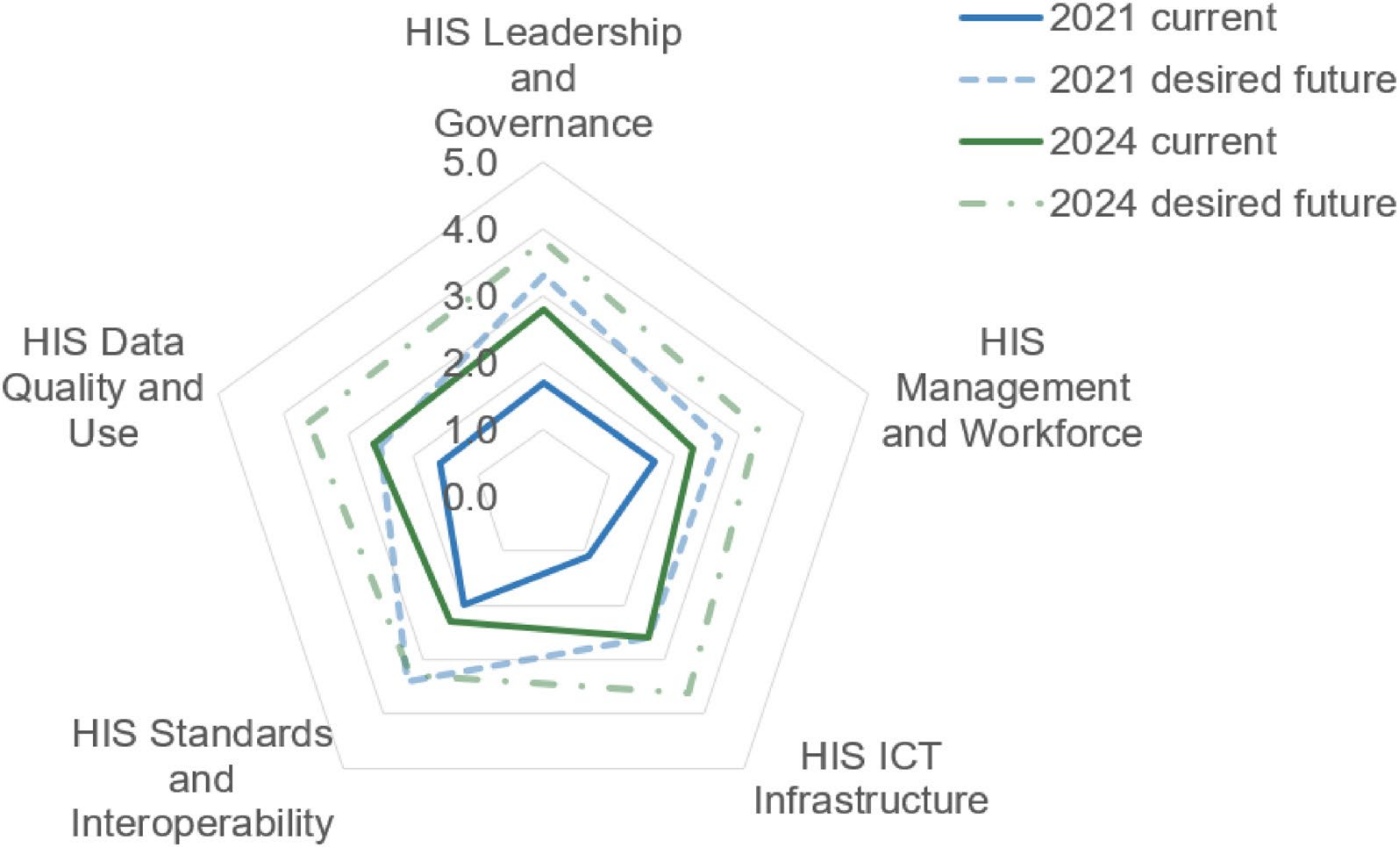
Current and future (desirable) scores of HIS maturity assessment on a 1–5 point scale, in 2021 and 2024. Interpretation of the scale: 1– emerging/ad hoc status; 2 – repeatable; 3 – defined; 4 – managed; and 5 – optimized

The average score of the current maturity assessment of HIS for all five domains in Serbia in 2024 had a value of 2.5, which is an increase of almost 1 full point compared with the situation in 2021 (“current 2021”), when it was 1.6 (Fig. 2). Progress was notable in all components and ranged from 0.3 to 2.5 points.

In 2021, the desirable maturity HIS status in the near future was 2.9. However, 3 years later, in 2024, it was not fully met, when the average score of the current state was 2.5 (Fig. 2). In 2024, desirable maturity HIS status in the future was 3.4, which is 0.9 points higher than the current state. This is exactly the same difference as between the current state in 2021 and 2024 (1.6 versus 2.5). Detailed results, i.e. points given to each subcomponent, and average scores per component and domain from the assessment of the current situation in 2021 and 2024 are provided in Supplementary File 2.

### HIS leadership and governance

**Fig. 3:**
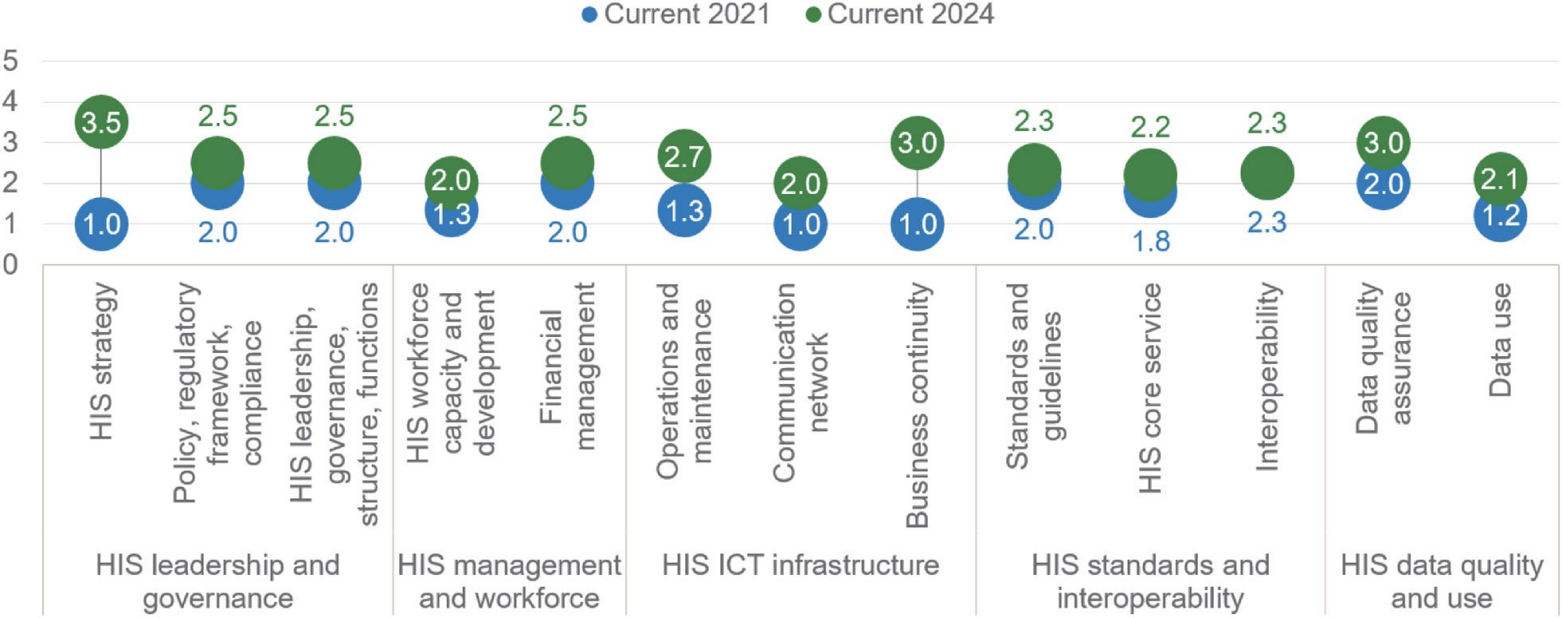
Average scores in the assessment of the current maturity state in 2024 and 2021

The greatest progress was achieved in the HIS Leadership and Governance domain, particularly the HIS strategy component, which increased from 1.0 to 3.5 (Fig. 3). This progress was justified by a number of facts and documents described below [20–22]. The most significant improvement in leadership and governance was adopting the eHealth Strategy for the period from 2022 to 2026 with an Action Plan for 2022–2023 [20–22]. This strategic document was launched 13 years after the previous one in this area, which provided an overall framework for establishing the HIS in Serbia [28]. The Action Plan 2022–2023 consisted of five objectives and approximately 100 measures and activities, the monitoring and status of implementation of which were meticulously conducted by NALED in an internal document that was the main source of information for this evaluation [22]. Further plans for monitoring and evaluation, as well as an overall HIS governance, will depend on the availability and decisions of the governmental body called Coordinating Body for Digitization in the Health System of the Republic of Serbia, which was appointed in 2023 [29] but has to be re-established, following the formation of the new government after the most recent elections [30].

HIS leadership and governance in the previous 3-year period was further strengthened by establishing an organizational unit within the Ministry of Health which is called the Sector for Governing Digitalization in the Health Sector, whose scope of activities is comprehensive and well defined [31].

The legislative and regulatory framework for digitalization has also been improved in the last 3 years by adopting the new Law on Health Documentations and Evidences in the Health Sector in 2023 [32]. This law provides a framework and directions for upscaling digitalization processes in the health sector in Serbia and its further development, including establishing *eKarton* (eHealth Records) and describing the elements, roles and responsibilities of the Republic Integrated Health Information System (RIZIS) [33].

### HIS management and workforce

In 2024, the domain HIS Management and Workforce was assessed 0.7 points higher than in 2021 (Fig. 2). It is mainly attributable to the realization of the eHealth Action Plan 2022–2023, which not only envisaged activities related to improving standards in IT knowledge and skills for using software solutions but also to ongoing activities of the Institute of Public Health of Serbia [20, 21], which is in charge of preparing practical instructions and guidelines for health professionals on how to use new functionalities of the online system for data collection in machine-readable form called *Servis javnog zdravlja* (Public Health Service) [34]. Higher points in the assessment of this component in 2024 were not given since Serbia still lacks an overall strategy for human resources development for health, although its development was envisaged 15 years ago in the strategic document called the “Decision on the plan for development of healthcare in Serbia” [35].

Strengthening workforce education will also happen by introducing multidisciplinary master studies in bioinformatics in 2025, which will be administered by the Faculty of Medicine University of Belgrade [36]. Therefore, it is expected that national workforce capacities will be improved in the future, and this justifies the higher points given for the future. In addition, in a draft Action Plan of the Program for Digitalization for the upcoming period, there is an objective related to the planning and development of human resources, knowledge and competencies for successful and sustainable digitalization in health services.

Gender equality in workforce development is also addressed in the SOCI evaluation of this domain [10]. Although gender equality was not mentioned in the eHealth Strategy and corresponding Action Plan, we identified other normative documents that regulate this topic. Thus, in the Republic of Serbia, gender equality is regulated by the Law on Gender Equality, adopted in 2021 [37]. Its Article 38 states that “gender equality in the field of ICT and information society includes, with special measures related to support for programs that contribute to the promotion of gender equality in the field of ICT and the deconstruction of gender stereotypes, the improvement of digital literacy and digital competences of women, and the promotion of the inclusion of young people, especially girls, in Science, Technology, Engineering and Mathematics (STEM) science and the ICT sector” [37]. Taking the following into consideration, it is expected that the HIS management and workforce will have gender mainstream activities in the future as there are set regulations for it.

### HIS ICT infrastructure

The average improvement in this domain over a 3-year period was 1.4 (Fig. 2). The highest increase of 2 points was reported for the component Business Continuity (Fig. 3). Business continuity has been improved by planning and creating health-related data migration from local information systems to the State Data Center for data management and storage [21, 22] and by connecting the local information systems of health institutions to the unified national ICT Network of Electronic Administration [38]. These solutions were preferable since they relied on already existing resources, such as governmental Office for IT and highly qualified experts employed there who could provide ongoing technical support to local health institutions. The challenge encountered during this process was related to the prioritization and selection of hospitals/healthcare institutions whose data would be migrated first. Building the Health Government Cloud in the national Data Center is a continuous process which is also depicted in the draft Action Plan for the future, and, therefore, it is expected to develop further and justify the points given for a higher level of maturity in the future.

### HIS standards and interoperability

The least progress in the 3-year period was reported for the HIS Standards and Interoperability domain (point difference of 0.3), in part because one of the components, Interoperability, remained at the same value as in 2021, 2.3 (Fig. 3). The subcomponents within this domain were already rated relatively high in the first assessment in 2021 (average rating 2.0), more than others, which is attributable to the existence of the already mentioned *Servis Javnog Zdravlja* from 2020 [4, 34]. Practical instructions and guidelines for its use were already developed by 2021, and it will be gradually expanded according to the needs envisaged in the recently adopted Law on Health Documentation and Records [32]. In Article 33, this law proposed establishing 14 different registers of diseases/health states with “bigger public health importance”, such as Registers for malignant diseases, diabetes mellitus, rare diseases, tuberculosis (TB), human immunodeficiency virus (HIV)/acquired immune deficiency syndrome (AIDS), among others. Registers are supposed to be populated with data in machine-readable format that is obtained from local HIS. Some registers are already functioning, while others remain to be created [32]. Serving that need, the Institute for Public Health of Serbia is developing unique methodological principles, standards, and procedures for interoperability, collecting and maintaining health documentation and records, as well as creating and submitting reports [23]. The Rulebook which should define the technical standards of the IT system, standards for local systems and interoperability (data exchange) is also being drafted, which will impose the standards for local systems on the basis of international standardized data formats and international recommendations and be in compliance with General Data Protection and Regulation, which is the national Law on Personal Data Protection, launched in 2018 [22]. HIS standards and interoperability will be improved by establishing *eKarton* (eHealth Records) as a minimal set of personal health data to be exchanged within the health system, which is an ongoing activity. Looking back at the period since 2021, the greatest challenges or factors that impeded further development in this domain were related to frequent changes of the decision-makers teams at the level of the ministry who had a different vision and preferences related to the content, structure and interoperability of *eKarton*.

Higher points at this domain were given from the perspective of the Agency for Medicines and Medical Devices (ALIMS), which has digitalized all its procedures and working processes, although it stated that interoperability with other important state institutions, such as the Health Insurance Fund, is currently missing and should be established [24, 25]. A significant number of ALIMS’s data sets are available on a state Open Data Portal [39].

### HIS quality of data and use of data

The Data Quality and Use domain showed that an average of 1.2 points progress was achieved in a 3-year period (Fig. 2). This is mainly owing to the creation of the legal and institutional framework for scaling up the exchange of data sets in a machine-readable format, between state-owned public health institutions, i.e. those which belong to the network of health institutions [7] and the already mentioned digital platform *Servis javnog zdravlja* [34]. Data management will be further improved by developing by-laws and rulebooks, according to the new Law on Health Documentation and Records in Health Sector, adopted in 2023 [32]. The network of regionally distributed public health institutes (*Zavodi za javno zdravlje*) is in charge of the quality control of data collected from local health institutions in their HIS. *Zavodi* are mandatory corrective mechanisms for assuring completeness and logical control of data.

The availability and use of data by citizens, in terms of accessing their own health-related data and scheduling appointments in the healthcare services at the primary level became possible through a mobile application and web portal that were developed since 2021 [40].

The subcomponent *Impact of Data Use* (within the component *Data Use*) received the most doubts in its interpretation by key informants, and it was rated with a modest score of 1 (not presented in the graph). Although the collected data is largely used for conducting different analyses and justification of decisions made, evaluation of the use of data or parameters for “measuring the impact of data use” are not defined. There is not an established system for measuring the impact of data use in the health system, which was perceived by key informants as one of the most sophisticated dimensions of HIS maturity.

The subcomponent *Data Synthesis and Communication* evaluates whether gender-segregated data are reported within national health statistics, and it is part of regular practice. Besides that, since 2005, gender-sensitive statistics have been periodically reported for a wide range of indicators, which are also informative for monitoring the achievement of Sustainable Development Goals [41].

## Discussion

This study aimed to find out what the level of HIS maturity was in the Republic of Serbia in 2024 compared with 2021, i.e. what was the progress made in the previous 3 years, using a system perspective and comprehensive approach, applying the previously tested SOCI method which combines desk review (objective and well-documented process) and consensus-based expert opinion to assign points of development.

The overall improvement of Serbian HIS in a 3-year period (2024 versus 2021) was 0.9 points (from 1.6 to 2.5, on a scale of 1–5). The components that showed the largest improvement were the HIS Strategic Plan (an increase of 2.5 in the Leadership and Governance domain) and Business Continuity (an increase of 2 in the ICT Infrastructure domain). Amongst the most important changes in the past 3 years was the adoption of the Program for Digitalization in the Health System in Serbia (i.e. eHealth Strategy) with the corresponding Action Plan, which provided a blueprint for development. These improvements are also reflected in the length of the SOCI report in 2021 (15 pages) and draft report in 2024 (40 pages) [42].

Positive lessons and improvements could be ascribed to the overall supportive context, which is characterized by a high level of governmental commitment, international donor support (mainly the European Union and USAID) and national availability of dedicated professionals in the field of IT, social medicine, informatics, and organizational and political sciences, which are leading to success stories. If the same eagerness and resources take place in the upcoming 3 years, and if the new draft of the eHealth Action Plan is adopted and implemented, it seems feasible to realize projected achievements (desired goals in the future), or improvements of 0.9 points, which means the HIS maturity could achieve a status between grade 3 and 4 (“defined” and “managed”). Otherwise, there is a threat of stagnation and missed opportunities for further development [43], which require significant investments that usually come from external sources of support, either credit, loans or donations. In combination with an insufficient political will to work towards the realization of the objectives of eHealth Strategy, the maturity status would remain the same as it is at the moment. However, by now, many important and innovative initiatives related to the use of IT at the national level have taken place, such as establishing the Institute for Artificial Intelligence Research and Development of Serbia [44] and launching (updating) a Strategy for Development of Artificial Intelligence in the Republic of Serbia for the period 2025–2030, after a previous one, for the period of 2020–2025 [45]. Both strategies clearly recognize health and healthcare as one of the fields that might benefit from an advanced HIS and artificial intelligence (AI). In addition, Serbia is appointed as a chair of the Global Partnership for Artificial Intelligence (GPAI) for the upcoming 3 years and hosted a GPAI summit in December 2024 where GPAI Belgrade Ministerial Declaration was adopted, which outlines key principles and priorities for future development and regulation of AI that align with ethical and human-centric values [46]. A promising initiative is Serbian accession to the “Program Digital Europe” in 2023, which is an opportunity for access to resources and application of new digital technologies [47].

Upcoming digital services will be *eKarton* (eHealthRecords) and *eBolovanje* (eSicknessLeave), whose development is ongoing. Their full implementation is supposed to benefit both individuals as citizens and their employers and will reduce costs of administrations and unnecessary transport and live contacts.

The application of the SOCI method in the evaluation of HIS in Serbia in 2024 recognized that different subsystems have different levels of development of information systems, with ALIMS leading the way. ALIMS fully digitized its business processes and made their datasets open to the public [25, 39]. However, the system as a whole is still fragmented, and interoperability between different subsystems remains a challenge. Data interoperability in standardized data formats (such as HL7, for example); high quality data collected through electronic health records; secured and scalable IT infrastructure and data governance and privacy concerns, are all important prerequisites that have to be optimally met to establish registers for diseases and enable a decision support system that relies on artificial intelligence, as found in a scoping review of Raycheva et al. [48]. The Institute of Public Health of Serbia is in charge of addressing these challenges by proposing to apply mandatory international standards for interoperability, with stable state funding, whereas policy-makers should define which are the institutions within an overall health system that are supposed to be networked. The optimal solution or model for informational networking of different institutions should be identified. The harmonization between systems is a subject of the needs assessment and priority setting, where economic analyses and availability of resources play an important role in the overall feasibility.

The process of maturity assessment conducted in Serbia directly contributed to the realization of the World Health Organization’s Global Strategy for Digital Health 2020–2025 and its Strategic Objective 2, which is to advance the implementation of national digital health strategies, with envisaged output such as “a developing or adopting dynamic digital health maturity assessment model” [49]. Applying the SOCI assessment method during two time periods represents a meaningful response by Serbia to the outline of this global strategy. 

Conducting maturity assessments is also in accordance with the Principles of Donor Alignment for Digital Health, endorsed by a broad coalition of international development partners, who recognized the need for the systematic use of maturity assessment tools in countries receiving external support for digital health initiatives [50]. These principles emphasize that comprehensive assessments should precede any donor-funded digital health investments, ensuring that such interventions are contextually appropriate, strategically aligned and capable of contributing to sustainable health system strengthening [50]. To our best knowledge, this is the first time that in one country two subsequent SOCI assessments are compared in one single academic paper, and in that sense, we strongly believe that conducting this analysis demonstrates a contribution to the sustainable health system strengthening and national capacities, which might encourage other countries with similar health information ecosystems to follow.

Apart from SOCI, there are many other tools for maturity assessment, such as the Digital Health Assessment Toolkit, the Global Digital Health Index (GDHI) and others [51]. At the moment of initiating maturity assessment process in 2021, national policy makers opted to use SOCI rather than a tool developed by the WHO, whose results (maturity score) are presented on a 0–100 point scale [52]. Our experiences with further use of the SOCI tool are related to the descriptions of points on a 1–5 Likert scale that are given in up to three sentences, which was not sufficient to comprehend and apply to the local context, and, therefore, subject to the individual and team interpretation. For future analyses we might recommend using the more narrow approach and evaluating one HIS component rather than overall governance in eHealth, especially since national strategic documents are in place, and digital governance achieved a higher level than at the beginning, in 2021.

## Limitations

A relative limitation is the fact that this maturity assessment did not cover local health information systems used in the private sector, since an integrated HIS in Serbia still does not include private healthcare providers, although there have been some initiatives to interconnect them as well. Private healthcare providers offer fragmented and mainly consultative healthcare services to those who can afford to pay mainly out of pocket, and their role in the integrated HIS would still be marginal. Although they are supposed to report their performance and health indicators equally as health facilities in public ownership, in reality, they are not subject to the scrutinized monitoring. 

Another relative limitation is the nature of this comprehensive maturity assessment itself, which is conducted at the system level, assessing system governance and accountability in defining the regulatory and operational framework for unlocking the full potential of HIS [4]. Therefore, this maturity assessment did not involve probing for end-user experiences (health professionals in urban and rural areas, or at different levels of healthcare) nor the opinion of citizens (including different population groups or patient advocacy groups) and their level of satisfaction. However, the process of creating the eHealth Strategy in 2021 was comprehensive and involved a wide range of stakeholders in the working group, including those mentioned above, whose perspectives were embedded in the eHealth Strategy.

While the other countries that conducted an HIS assessment using the SOCI tool do not have a similar historical, geopolitical and sociodemographic context with Serbia, there are some commonalities. In particular, the other countries where two measurement points are available show improvements in HIS leadership and governance and data quality and use. This demonstrates the feasibility and acceptance of the interventions implemented to address gaps identified in the assessment and that HIS governance is a precursor for progress in other areas such as data use. [12–19]. We consider the SOCI method as an excellent tool for stirring discussion, sharing knowledge and different perspectives of key informants, human capacity building, and fulfilling identified gaps in planning [4, 53, 54]. It remains to be seen in the future whether the same pace of improvements will be preserved and what the factors are that are contributing to its success or unaddressed challenges.

## Supplementary Information

Additional File 1.

Additional File 2.

## Data Availability

No datasets were generated or analysed during the current study.
