# Supplementary material for: Serbian Health Information System (HIS) improvements 2021–2024: comparison study using stages of continuous improvement (SOCI) methodology
Source: Health Res Policy Syst. 2025 Jul 14;23:92. doi: 10.1186/s12961-025-01337-5 (PMC12257858; doi:10.1186/s12961-025-01337-5)
Supplement: Supplementary file 2 — Additional File 2. [file 12961_2025_1337_MOESM2_ESM.docx]

**Supplement 1.** HIS Stages of Continuous Improvement: Core Domains, HIS Components and HIS Subcomponents, and corresponding points and average scores, on a 1 to 5 scale, with a following interpretations: 1-Emerging/Ad hoc; 2- Repeatable; 3-Defined; 4- Managed; and 5-Optimized .

| **HIS Core Domains** | **Average score per domain 2021 vs. 2024** | **HIS Components** | **Average score per compon. 2021** | **Average score per compon. 2024** | **HIS Subcomponents** | **Point 1-5**  **2021** | **Point 1-5**  **2024** |
| --- | --- | --- | --- | --- | --- | --- | --- |
| **Leadership and Governance** | 1.7 vs. 2.8 | HIS strategic plan or HIS strategy | 1.0 | 3.5 | HIS strategic plan | 1 | 4 |
|  |  |  |  |  | Monitoring and Evaluation Plan | 1 | 3 |
|  |  | Policy, legal and regulatory framework and compliance | 2.0 | 2.5 | Existence of HIS policies and legislation | 2 | 3 |
|  |  |  |  |  | Policy compliance enforcement | 2 | 2 |
|  |  | HIS leadership and governance organizational structures and functions | 2.0 | 2.5 | HIS leadership and coordination | 2 | 3 |
|  |  |  |  |  | HIS organizational structure and functions | 2 | 2 |
| **HIS management and workforce** | 1.6 vs. 2.2 | HIS workforce capacity and development | 1.3 | 2.0 | HIS competencies (knowledge, skills and abilities) | 2 | 2 |
|  |  |  |  |  | HIS training and education (include continuous professional development) | 1 | 2 |
|  |  |  |  |  | HR policy | 1 | 2 |
|  |  | Financial management | 2.0 | 2.5 | HIS financing plan | 2 | 3 |
|  |  |  |  |  | Resource mobilization | 2 | 2 |
| **HIS ICT infrastructure** | 1.2 vs. 2.6 | Operations and maintenance | 1.3 | 2.7 | Reliable power/electricity | 1 | 2 |
|  |  |  |  |  | ICT business infrastructure support | 1 | 3 |
|  |  |  |  |  | Hardware | 2 | 3 |
|  |  | Communication network (LAN and WAN) | 1.0 | 2 | Network and internet connectivity | 1 | 2 |
|  |  | Business continuity | 1.0 | 3 | Business continuity processes and policies | 1 | 3 |
| **HIS standards and inter-operability** | 2.0 vs. 2.3 | Standards and guidelines | 2.0 | 2.3 | HIS standards and guidelines | 2 | 2 |
|  |  |  |  |  | Data set definitions (clinical, laboratory, commodities and indicator) | 2 | 3 |
|  |  |  |  |  | Data exchange standards | 2 | 2 |
|  |  | HIS core services | 1.8 | 2.2 | Master facility list | 3 | 3 |
|  |  |  |  |  | Indicator registry | 1 | 2 |
|  |  |  |  |  | Terminology management | 2 | 2 |
|  |  |  |  |  | Unique person identity management | 2 | 2 |
|  |  |  |  |  | Enterprise architecture | 1 | 2 |
|  |  | Interoperability (data exchange) | 2.3 | 2.3 | Person data exchange | 2 | 2 |
|  |  |  |  |  | Aggregate data exchange | 2 | 2 |
|  |  |  |  |  | Community management data exchange | 2 | 2 |
|  |  |  |  |  | Data security exchange | 3 | 3 |
| **HIS data quality and use** | 1.4 vs. 2.3 | Data quality assurance | 2.0 | 3.0 | Data quality assurance and quality control | 2 | 3 |
|  |  |  |  |  | Data management | 2 | 3 |
|  |  | Data use | 1.2 | 2.1 | Data use availability strategy | 1 | 2 |
|  |  |  |  |  | Information/ data availability | 1 | 2 |
|  |  |  |  |  | Data use competencies | 1 | 2 |
|  |  |  |  |  | User/ stakeholder engagement | 1 | 2 |
|  |  |  |  |  | Data synthesis and communication | 2 | 2 |
|  |  |  |  |  | Reporting and analytics features | 2 | 3 |
|  |  |  |  |  | Data use impact | 1 | 1 |
|  |  |  |  |  | Data collection alignment with workflow | 1 | 3 |
|  |  |  |  |  | Decision support (clinical or other) | 1 | 2 |

Source: updated from Ollis et al, 2024 (4).
